# Influence of family members on utilization of maternal health care services among teen and adult pregnant women in Kathmandu, Nepal: a cross sectional study

**DOI:** 10.1186/1742-4755-11-92

**Published:** 2014-12-23

**Authors:** Priti Upadhyay, Tippawan Liabsuetrakul, Amir Babu Shrestha, Neelam Pradhan

**Affiliations:** Epidemiology Unit, Faculty of Medicine, Prince of Songkla University, Hat Yai, Songkhla 90110 Thailand; Paropakar Maternity and Women’s Hospital, Thapathali, Kathmandu Nepal; Department of Obstetrics and Gynecology, Tribhuban University Teaching Hospital, Maharajgunj, Kathmandu Nepal

**Keywords:** Influence, Family members, Husband, Mother-in-law, Utilization, Maternal health care services, Antenatal care, Delivery, Teens, Adults

## Abstract

**Background:**

In some developing countries a woman’s decision to utilize maternal health care services is not made by the woman herself but by other family members. The perception of family members regarding who is the most influential person for making the decision to utilize these services is inconclusive. Hence, this study aimed to determine the perceived influential person on utilization of antenatal care (ANC) and delivery care services among teen, young adult and adult pregnant women from the perspective of the woman themselves, their husband and their mother-in-law, identify the factors associated with the woman being the most influential person, and assess the level of agreement between the woman’s and her husband’s response to the woman being the most influential person.

**Methods:**

A cross-sectional study was conducted at Paropakar Maternity and Women’s Hospital and Tribhuvan University Teaching Hospital. Purposive sampling technique was used to select 315 women of which 105 were from each age group and their accompanied husbands (n = 315) and mothers-in-law (n = 315). The proportion of perceived influential person and mean priority score of the perceived influence with its 95% confidence interval was calculated. The factors associated with the woman perceived as the most influential person were analyzed by multivariate logistic regression model. The agreement was analyzed using kappa statistic.

**Results:**

Among teens and young adults and their husband and mother-in-law, the woman’s husband was perceived as the most influential person. Among adults, the most influential person for ANC was the woman herself but for delivery care was the woman’s husband. A woman of adult age, having a non-indigenous ethnicity or who was not referred was more likely to perceive herself as the most influential person in the decision to utilize delivery care. A fair to poor level of agreement was found on the perception of the most influential person for ANC and delivery care utilization.

**Conclusions:**

Both women and their husbands influenced the decision to utilize ANC and delivery care but husbands were more influential, especially in teens and young adults. Thus, husband’s involvement is crucial as a strategy to improve maternal health care utilization in Nepal.

**Electronic supplementary material:**

The online version of this article (doi:10.1186/1742-4755-11-92) contains supplementary material, which is available to authorized users.

## Background

In 2010, 99% of all global maternal deaths occurred in developing countries [1]. Compared to women aged over 20 years, the risks of maternal death among teens aged 15–19 years is twice as high and is almost five times higher among those under 15 years [2]. Likewise, the risk of stillbirth and neonatal death in the first week of life is higher among newborns of mothers who are aged younger than 20 years [3]. Maternal and perinatal deaths and related complications can be averted by timely and adequate utilization of antenatal care (ANC) and delivery care services [4, 5].

Low utilization of maternal health care services and increased risks for obstetric complications among pregnant teens has been reported from both developed and developing countries [6–10]. As a result, teens are a vulnerable group and need more attention for their care seeking behaviors when they become pregnant. In developing countries in Africa and Asia, particularly Nepal, low utilization of maternal health care services was observed, among teens and adults [11–13].

The proportion of births attended by skilled health personnel and ANC coverage (the percentage of women aged 15-49 with a live birth that received ANC by any provider four or more times during their pregnancy), two of the WHO indicators for improving maternal and child health, have improved globally [14]. Although the government of Nepal implemented the safe delivery incentive program in 2005 and a national free delivery policy in 2009, Nepal has not achieved the Millenium Development Goal 5 (MDG5) targets for these indicators yet. The latest national data in 2013 showed that only 50% of women had achieved both MDG 5 targets while the target to meet by 2015 is 80% for four ANC visits and 60% for delivery attended by skilled birth attendant [15–17]. A higher utilization of facility-based services was evident in semi-urban regions of Kathmandu district with 70% of deliveries attended by a skilled birth attendant. Lower utilization in remote regions of Nepal might be explained by the cultural tradition of giving birth in the community and women’s lack of power to demand the services if their partners show discouragement [18, 19].

It is evident that the factors associated with utilization of obstetric care in facilities in developing countries such as Nepal are multifactorial and include demographic, economic, socio-cultural or religious characteristics [20–23]. Apart from the women’s individual and household factors, it was reported in Africa and South Asia that the decision to utilize maternal health care services was not made by the woman herself but by her husband or other family members [3, 24, 25]. Six previous studies conducted in Nepal, Bangladesh, Burkina Faso, Thailand, Kenya and Pakistan determined the influential persons for making the decision to utilize maternal care; however, the study samples, methodologies and data analyses were different to ours [24–29].The study from Nepal was qualitative and interviewed women, their husbands and their mothers-in-law [24]. Four studies interviewed only women among which two were qualitative and two were quantitative [25, 27–29]. The remaining one was also a qualitative study which interviewed both women and their husbands [26]. The findings of qualitative studies were limited by the small sample size. In addition, the methodologies applied to identify the most influential person were not clearly explained in the previous studies. Additionally, previous studies on household decision making have revealed that the women’s autonomy was positively associated with age suggesting that the influential persons might vary depending on age [30, 31].

Increasing the effort to improve ANC coverage and the proportion of births attended by skilled birth attendants is a challenge in the countries of Africa and South Asia. The efforts may be useless if the relevant persons who participate in the decision making process are not taken into account. Therefore, there is a need to identify the influential person on making decision to utilize these services. Knowing the influential persons among the family members of the pregnant women and in different age groups is essential to prepare the strategies and targets of promotion of maternal health care services. This study thus aimed to determine the perceived influential person on a woman’s decision to utilize antenatal and delivery care services among teen, young adult and adult pregnant women from the perspective of themselves, their husband and their mother-in-law, identify the factors associated with the woman being the most influential person in the decision to utilize care, and assess the level of agreement between the woman’s and husband’s response to the woman being the most influential person.

## Methods

### Study design and setting

A cross-sectional study was conducted at Paropakar Maternity and Women’s Hospital (PMWH) and Tribhuvan University Teaching Hospital (TUTH) which provides maternity services in Kathmandu district of Nepal, from June to October 2013. The PMWH is the main public maternity hospital of Kathmandu having more than 18,000 deliveries per year and the TUTH is a university hospital having more than 5,000 deliveries per year in 2013.

### Study sample

Women aged 34 or less, who delivered at PMWH or TUTH and were accompanied by both their husband and mother-in-law at any time of their hospitalization were purposively included. The sample also consisted of the accompanying husband and mother-in-law. Eligible women, their husbands and mothers-in-law were mostly identified at the postpartum ward by asking the women whether her husband and mother-in-law had accompanied her or would visit her at this admission. Some women were also identified when the woman’s husband or mother-in-law were waiting outside the labor room. All women, their husbands and mothers-in-law were informed that they had the right to agree or refuse to participate in the study and each of them was approached separately and independently.

The sample size was calculated on the basis of estimating the proportion of women whose family members influenced her to utilize ANC and delivery care services. Considering this estimated proportion to be 50%, to be 95% confident of estimating this proportion with a precision of 10%, at least 96 women were needed. We assumed that the influence of family members on women of different age groups may not be same, thus the sample size was separated into three age groups: teens (less than 20 years), young adults (20-24 years) and adults (25-34 years). As a result, at least 288 women and their husbands and mothers-in-law were required. Sample sizes were determined for each hospital based on the probability proportional to the number of deliveries in each hospital. The proportion of past deliveries was approximately 70% and 30% in PMWH and TUTH respectively, which lead to the sample size of 205 for PMWH and 110 for TUTH.

### Data collection

After individually signing the consent forms, all respondents were interviewed separately in a private place within the hospital using a structured questionnaire. The interviews of the women were done exclusively in the postpartum period before the women were discharged from the hospital. The interviews of the husbands and mothers-in-law were performed in either the postpartum or delivery period as appropriate. All interviews were conducted by the first author. All women, husbands and mothers-in-law were interviewed using the same questionnaire.

### Variables

The main outcome variable was the person who had the most influence on the woman’s decision to utilize ANC and delivery care at the hospital. The question asked to all three persons was “When you/your spouse/your daughter-in-law needed ANC /delivery care for this pregnancy, who were the influential persons on your/your wife’s/your daughter-in-law’s decision to utilize this care?” with four possible persons, namely the woman, husband, mother-in-law and others. If they responded multiple answers then they were asked to rank the priority of each influential person from the most to the least with no ties allowed. The person who was ranked the highest for each respondent was defined as the most influential person as either the woman or others.

Independent variables included socio-demographic characteristics of the women, husband and mother-in-law and obstetric characteristic of the women.

### Statistical analysis

Double data entry and validation was performed using EpiData. The analysis was done in R (The R Foundation for Statistical Computing, Vienna, Austria). The characteristics of women, their husbands and their mothers-in-law were analyzed using Chi square or Fisher’s exact test as appropriate for categorical variables and Wilcoxon’s Rank Sum test for continuous variables.

The proportion of each influential person by the three types of respondents and three age groups for both ANC and delivery care was determined and the mean priority score was calculated with 95% confidence interval. The priority ranks were summed and then averaged for each person. Higher mean scores indicated a greater influence of that person on the woman’s decision to utilize ANC and delivery care.

The factors associated with women being perceived as the most influential person on the decision to utilize ANC and delivery care by women’s perspective were assessed by uni-variate and multiple logistic regression models. A p-value less than 0.05 was considered as statistically significant. Agreement between women’s and their husbands’ perspectives on whether the woman was the most influential person on the utilization of both ANC and delivery care was analyzed using both unadjusted and prevalence adjusted kappa statistic among the three age groups [32].

### Ethical considerations

The study protocol was approved by the Ethical Committee of the Faculty of Medicine, Prince of Songkla University, Songkhla, Thailand. The permission for conducting data collection in the participating hospitals was obtained. All respondents were provided with information about the study, and written informed consent was received before the interview. Involvement in the study did not place the respondents at any risk. Data were collected anonymously to ensure confidentiality.

## Results

A total of 315 women with their husbands and mothers-in-law were interviewed in the study period of which 105 in each age group were included. The socio-demographic and obstetric characteristics of women are presented in Tables 1 and 2 respectively. There were a significantly higher proportion of indigenous teens in the study sample. A significantly higher proportion of teens also resided outside of Kathmandu valley and had no formal occupation. They had a lower proportion who had attained a higher educational degree and had their marriage arranged by their family. A higher proportion of teens attended their first ANC after four months gestation, had less than four ANC visits and had a low perceived willingness to utilize ANC.

**Table 1:** **Comparison of women’s socio-demographic characteristics of by age group**

Characteristics	Teens (N = 105) n (%)	Young adults (N = 105) n (%)	Adults (N = 105) n (%)	Total (N = 315) n (%)	P-value
**Ethnicity**					0.02
Indigenous	72 (68.6)	55 (52.4)	56 (53.3)	183 (58.1)	
Non-indigenous	33 (31.4)	50 (47.6)	49 (46.7)	132 (41.9)	
**Religion**					0.08
Hindu	80 (76.2)	90 (85.7)	91 (86.7)	261 (82.9)	
Buddhist or others	25 (23.8)	15 (14.3)	14 (13.3)	54 (17.1)	
**Residency**					0.001
Within Kathmandu	82 (78.1)	89 (84.8)	100 (95.2)	271 (86)	
Outside Kathmandu	23 (21.9)	16 (15.2)	5 (4.8)	44 (14.0)	
**Occupation**					<0.001
Housewife	78 (74.3)	56 (53.3)	51 (48.6)	185 (58.7)	
Employed	8 (7.6)	37 (35.2)	44 (41.9)	89 (28.3)	
Farmer or laborer	19 (18.1)	12 (11.4)	10 (9.5)	41 (13.0)	
**Education**					<0.001
Illiterate or can only read and write	7 (6.7)	4 (3.8)	13 (12.4)	24 (7.6)	
Primary or secondary	90 (85.7)	75 (71.4)	65 (61.9)	230 (73.0)	
University degree or higher	8 (7.6)	26 (24.8)	27 (25.7)	61 (19.4)	
**Marriage arrangement**					<0.001
By self or partner	73 (69.5)	57 (54.3)	42 (40)	172 (54.6)	
By family	32 (30.5)	48 (45.7)	63 (60)	143 (45.4)	
**Age at marriage**					<0.001
Less than 20 years	105 (100)	50 (47.6)	18 (17.1)	173 (54.9)	
More than or equal to 20 years	-	55 (52.4)	87 (82.9)	142 (45.1)

**Table 2:** **Comparison of women’s obstetric characteristics of by age group**

Characteristics	Teens (N = 105) n (%)	Young adults (N = 105) n (%)	Adults (N = 105) n (%)	Total (N = 315) n (%)	P-value
**Parity**					<0.001
Primiparous	98 (93.3)	86 (81.9)	45 (42.9)	229 (72.7)	
Multiparous	7 (6.7)	19 (18.1)	60 (57.1)	86 (27.3)	
**Gravida**					<0.001
Primigravida	88 (83.8)	75 (71.4)	37 (35.2)	200 (63.5)	
Multigravida	17 (16.2)	30 (28.6)	68 (64.8)	115 (36.5)	
**Intention of current pregnancy**					0.067
Not sure/No	45 (42.9)	29 (27.6)	36 (34.3)	110 (34.9)	
Yes	60 (57.1)	76 (72.4)	69 (65.7)	205 (65.1)	
**Type of hospital for ANC**					0.002
Only Public	93 (88.6)	83 (79.0)	77 (73.3)	253 (80.3)	
Only Private	7 (6.7)	7 (6.7)	3 (2.9)	17 (5.4)	
Both public and private	5 (4.8)	15 (14.3)	25 (23.8)	45 (14.3)	
**Gestational age at first ANC**					0.02
Within four months	59 (56.2)	75 (71.4)	75 (71.4)	209 (66.3)	
After four months	46 (43.8)	30 (21.6)	30 (21.6)	106 (33.7)	
**Number of ANC visits**					0.003
Less than four times	16 (15.2)	2 (1.9)	10 (9.5)	28 (8.9)	
More than four times	89 (84.8)	103 (98.1)	95 (90.5)	287 (91.1)	
**Referral status**					0.008
Not referred from another hospital	88 (83.8)	89 (84.8)	101 (96.2)	278 (88.3)	
Referred from another hospital	17 (16.2)	16 (15.2)	4 (3.8)	37 (11.7)	
**Perceived willingness to utilize ANC**					0.03
Low	26 (24.8)	12 (11.4)	17 (16.2)	55 (17.5)	
High	79 (75.2)	93 (88.6)	88 (83.8)	260 (82.5)	
**Perceived willingness to utilize delivery care**					0.9
Low	12 (11.4)	10 (9.5)	11 (10.5)	33 (10.5)	
High	93 (88.6)	95 (90.5)	94 (89.5)	282 (89.5)	

**Figure 1:**
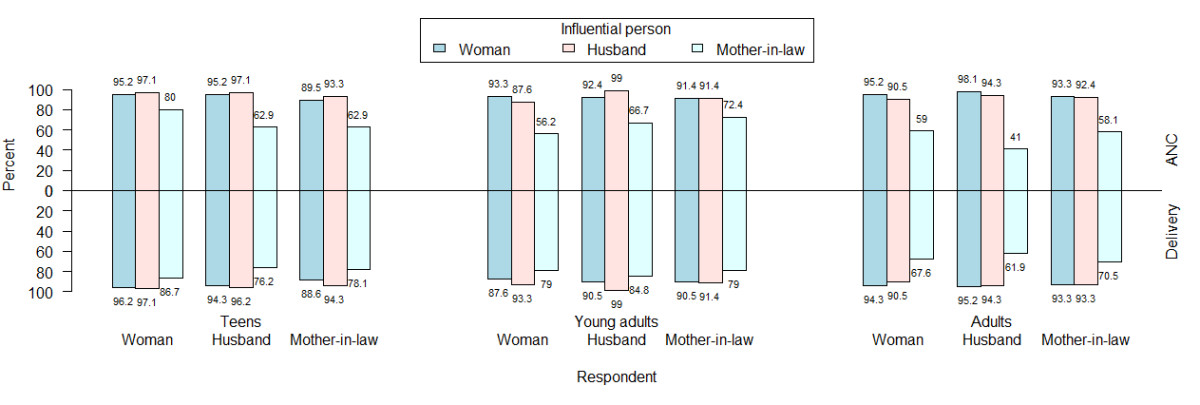
**Proportion of influential persons rated by three types of respondents on the decision to utilize ANC and delivery care by women’s age group.**

**Figure 2:**
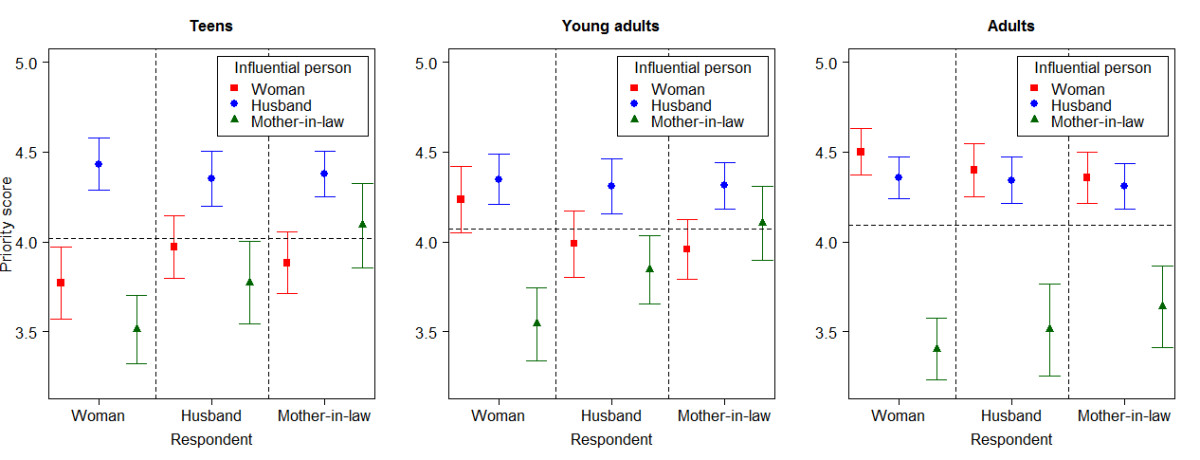
**Comparison of mean priority scores for influential persons on decision to utilize ANC services by age groups.**

**Figure 3:**
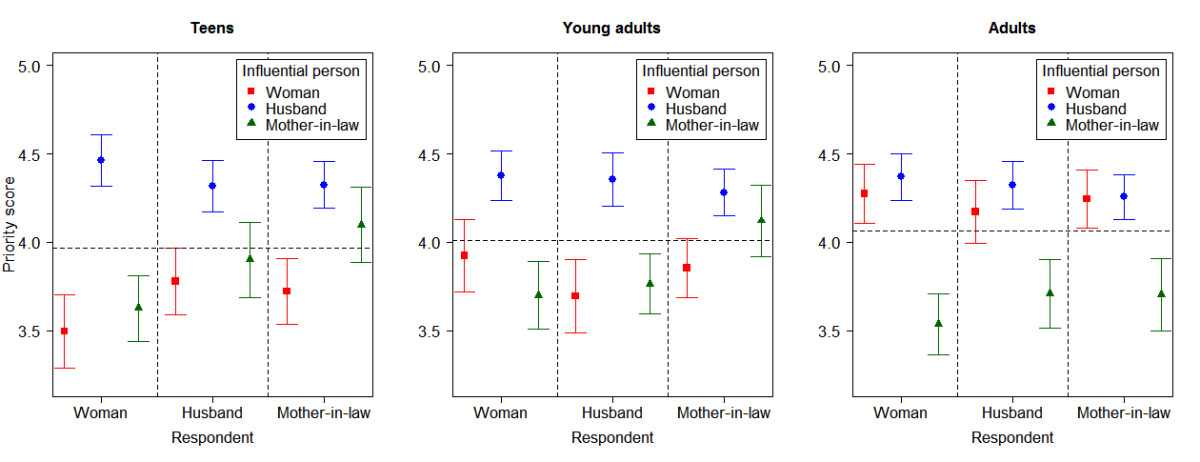
**Comparison of mean priority scores for influential persons on decision to utilize delivery care services by age groups.**

Table 3 compares the characteristics of the women’s husbands and mothers-in-law. The median ages of husbands and mothers-in-law increased with increasing age group. Husbands of teens had a lower proportion of higher educational attainment and employment. The majority (>80%) of mothers-in-law had not attended formal education regardless of the age of the women. The proportion of housewives among the mothers-in-law of adult women was twice as high as that of young adults and teens. Slightly more than half of mothers-in-law currently lived with their daughter-in-law.Figure 1 shows the proportion of influential persons perceived by the three types of respondents on the decision to utilize facility-based ANC and delivery care stratified by the women’s age groups. Approximately 90% or more of women and their husbands and mothers-in-law in all three age groups rated that the woman and her husband were influential persons in the decision to utilize both services. Mothers-in-law were rated to be the lowest influential person by three types of respondents for both services, especially in the adult group and its proportion of influence was lower in ANC than in delivery care.The mean priority scores of women, husbands and mothers-in-law as the influential person in the decision to utilize ANC and delivery care rated by three types of respondents among three age groups are shown in Figures 2 and 3. For ANC (Figure 2), the husband was ranked to be the highest among teens and young adults. Among adults, women were ranked the highest followed closely by husbands. Both women and husbands perceived that the woman was more influential than the mother-in-law across all age groups but the mother-in-law perceived her influence on her daughter-in-law’s utilization of ANC among teens and young adults. For delivery care (Figure 3), the husband was also ranked as the most influential person in all age groups. The influence of women in their decision to utilize delivery care was ranked the lowest by all three respondents among the teen age group.

**Table 3:** **Characteristics of husbands and mothers-in-law by women’s age group**

Characteristics	Teens (N = 105) n (%)	Young adults (N = 105) n (%)	Adults (N = 105) n (%)	Total (N = 315) n (%)	P-value
**Husband’s characteristics**					
**Age**					
Median (IQR)	23 (21,24)	26 (24,29)	30 (28,33)	26 (23,30)	<0.001*
**Education**					<0.001**
Illiterate or can only read and write	3 (2.9)	1 (0.9)	4 (3.8)	8 (2.5)	
Primary or secondary	92 (87.6)	77 (73.3)	64 (60.9)	233 (73.9)	
University degree or higher	9 (8.6)	27 (25.7)	37 (35.2)	74 (23.2)	
**Occupation**					0.02**
Unemployed	5 (4.8)	4 (3.8)	3 (2.9)	12 (3.8)	
Employed	60 (57.1)	79 (75.2)	79 (75.2)	218 (69.2)	
Farmer or others	40 (38.1)	22 (21)	23 (21.9)	83 (26.3)	
**Type of family**					0.2
Nuclear	33 (31.4)	40 (38.1)	45 (42.9)	108 (34.3)	
Joint	72 (68.6)	65 (61.9)	60 (57.1)	197 (62.5)	
**Per capita income**					0.7
< 700 USD	72 (68.6)	73 (69.5)	68 (64.8)	213 (67.6)	
≥ 700 USD	33 (31.4)	32 (30.5)	37 (35.2)	102 (32.4)	
**Mother-in-law’s characteristics**					
**Age**					<0.001*
Median (IQR)	45 (42,50)	50 (45,55)	55 (50,60)	50 (45,55)	
**Education**					0.2
Illiterate or can only read and write	88 (83.8)	87 (82.9)	95 (90.5)	270 (85.7)	
Secondary or lower	17 (16.2)	18 (17.1)	10 (9.5)	45 (14.3)	0.2
**Occupation**					<0.001
Housewife	25 (23.8)	26 (24.8)	52 (49.5)	103 (32.7)	
Farmer or laborer	68 (64.8)	63 (60)	48 (45.7)	174 (55.2)	
Employed	12 (11.4)	16 (15.2)	5 (4.8)	33 (10.5)	
**Lives with daughter-in-law**					0.09
No	43 (41)	39 (37.1)	54 (51.4)	136 (43.2)	
Yes	62 (59)	66 (62.9)	51 (48.6)	179 (56.8)	

*Wilcoxon’s Rank Sum test **Fisher’s exact test.

Of all women, 44% and 33% of them perceived themselves to be the most influential person for making the decision to utilize ANC and delivery care respectively. Table 4 presents the factors associated with the woman being the most influential person in the decision to utilize ANC and delivery care. Women’s age group, ethnicity and referral status were significant factors for utilization of ANC while age group and ethnicity were the only significant factors for delivery care utilization. For ANC, young adult females and adult females, compared to teen females, perceived that they were the most influential person to make the decision (Adjusted odds ratio (AOR) 2.12; 95% Confidence interval (CI) 1.18-3.79 for young adults and AOR 3.14; 95% CI 1.76-5.63 for adults). For delivery care, adult females were more likely to perceive themselves to be the most influential person (AOR 2.9; 95% CI 1.56-5.38). Non-indigenous women perceived that they were the most influential person to make the decision for both ANC (AOR 1.77; 95% CI 1.11-2.83) and delivery care (AOR 1.71; 95% CI 1.04-2.8). For delivery care, those who were not referred from other hospitals were more likely to perceive themselves to be the most influential person compared to those who were referred from other hospitals (AOR 3.77; 95% CI 1.27-11.15).

**Table 4:** **Final logistic regression model identifying factors associated with women being perceived as the most influential person on decision to utilize ANC delivery care**

Factors	ANC			Delivery		
	N = 315			N = 315		
	AOR (95% CI)	P-value^*^	P-value^**^	AOR (95% CI)	P-value^*^	P-value^**^
**Age group of women:** ref. = Teens			<0.001			0.002
Young adults	2.12 (1.18,3.79)	0.01		1.54 (0.81,2.93)	0.2	
Adults	3.14 (1.76,5.63)	<0.001		2.9 (1.56,5.38)	<0.001	
**Ethnicity of women:** ref. = Indigenous Non-Indigenous	1.77 (1.11,2.83)	0.02	0.02	1.71 (1.04,2.8)	0.03	0.03
**Referral status:** ref. = Referred Not referred				3.77 (1.27,11.15)	0.02	0.006

AOR Adjusted odds ratio, CI Confidence interval, *Wald’s test, ** Likelihood ratio test

Factors included in the initial model for ANC decision-making were age at marriage of women, type of marriage arrangement, age and occupation of mother-in-law, and daughter-in-law and mother-in-law sharing same residence.

Factors included in the initial model for delivery decision-making were place of residence, occupation and age at marriage of women, place of ANC, gestational age at first ANC, willingness to attend ANC, education level of husband, per capita income, age and occupation of mother-in-law.

Table 5 presents the agreement between the woman and her husband on the most influential person on the woman’s decision to utilize ANC and delivery care. The observed agreement ranged from 55.2% to 60% for ANC and 55.2% to 66.7% for delivery care utilization. Fair to poor agreement (prevalence-adjusted kappa coefficient 0.1 to 0.3) was found for both services of which poor agreement was observed among adults. The proportion of other persons rated as the most influential person by both husband and women was detected among teens (51.4%), young adults (40.0%) and adults (22.8%) for ANC and among teens (60.0%), young adults (55.2%) and adults (31.4%) for delivery care.

**Table 5:** **Agreement of the most influential person for ANC and delivery care by age groups**

	Antenatal care					Delivery care				
	Husband’s response					Husband’s response				
Teen’s response		Woman (%)	Other (%)	Total (%)			Woman (%)	Other (%)	Total (%)	
	Woman	9 (8.6)	20 (19.0)	29 (27.6)	K = 0.02	Woman	7 (6.7)	15 (14.3)	22 (21)	K = 0.07
	Other	22 (20.9)	54 (51.4)	76 (72.3)	Kpa = 0.2	Other	20 (19.0)	63 (60)	83 (79)	Kpa = 0.3
	Total (%)	31 (29.5)	74 (70.4)	105 (100)		Total (%)	27 (25.7)	78 (74.3)	105 (100)	
Young adult’s response										
	Woman	19 (18.8)	30 (28.6)	49 (46.7)	K = 0.1	Woman	9 (8.6)	23 (21.9)	32 (30.5)	K = 0.08
	Other	14 (13.3)	42 (40)	57 (54.3)	Kpa = 0.2	Other	15 (14.3)	58 (55.2)	73 (69.5)	Kpa = 0.3
	Total (%)	33 (31.4)	72 (68.6)	105 (100)		Total (%)	24 (22.9)	81 (77.1)	105 (100)	
Adult’s response										
	Woman	34 (32.4)	25 (23.8)	59 (56.2)	K = 0.1	Woman	25 (23.8)	25 (23.8)	50 (47.6)	K = 0.1
	Other	22 (21)	24 (22.8)	46 (43.8)	Kpa = 0.1	Other	22 (21)	33 (31.4)	55 (52.4)	Kpa = 0.1
	Total (%)	56 (53.3)	59 (56.2)	105 (100)		Total (%)	47 (44.8)	58 (55.2)	105 (100)	

K: kappa coefficient; Kpa: prevalence-adjusted kappa coefficient.

## Discussion

In our study, women, along with their husbands and mothers-in-law, all perceived that the husband was the most influential person in the woman’s decision to utilize ANC and delivery care, particularly in teens and young adults. In contrast, adult women, along with their husbands and mothers-in-law perceived the woman to be the most influential person for utilization of ANC services. A woman aged 25-34, having a non-indigenous ethnicity or who was not referred from another hospital was more likely to perceive herself as the most influential person for making the decision to utilize delivery care services. The level of agreement between a woman and her husband on the most influential person was poor to fair.

In our study, a high proportion of the women and their husbands were perceived to be influential person but the husband was found to be more influential based on our ranking technique. The influential person on the decision to utilize maternal health care services differs among countries and might be explained by the differences in cultural, social and health system contexts as well as variations in the measurement technique. The influence of the husband as the main decision maker for a woman’s utilization of maternal health services found in this study was also found in previous studies in Bangladesh, Burkina Faso, Tanzania and Nepal [24–26, 33]. However, a study in Thailand found that the women themselves influenced their own decision on utilization of delivery care more than other family members [27]. In Nepal and other South Asian and African countries which have patriarchal societies, males are prominent in making the decisions [24, 34]. This may be related to being a household head and financial authority as approximately 88% of women in our study said that the hospital expenditure was paid by their husband (data not shown). On the other hand, in Thailand, gender equality [35] and universal health coverage [36] have given women more empowerment.

In addition to the couple, the mother-in-law could be a person who influences the decision to utilize maternal health care services [24, 25]. A community-based study in Mali found that the traditional belief by the mother-in-law of home delivery influenced the place of delivery [34]. However, the influence of the mother-in-law was not as prominent as the husband’s or woman’s influence in our study. This might be related to the study setting being an urban area while the previous three studies were conducted in rural areas where the traditional society persists and imparts greater power to the mother-in-law in the management of household and health decisions [25, 37, 38]. Nuclear families are free from the influence of mothers-in-law, who do not live in the same household and this is prominent in urban settings. The explanation of more influence of the mother-in-law in delivery care than ANC could not be explained but it may be related to more traditional beliefs in delivery care than in ANC.

The proportion of women perceiving themselves as the most influential person in the decision to utilize delivery care in our study was lower than reported from a study in Kenya but higher than that from a study in Pakistan. However, in both studies the assessment of the decision maker was done differently than in our study [28, 29]. Adult women were more independent in decision making which was supported by two studies conducted in South Asian countries and age was positively associated with the household decision-making autonomy [30, 31]. In a study from Tanzania, teen pregnant women were more likely to receive advice from their mother or a close person rather than from their partner in comparison to adult women [12]. A report from the World Health Organization (WHO) also mentioned that young women including teens had a lack of authority for decision making or resources for using health services [39]. Women with non-indigenous ethnicity belong to an upper social class in Nepal and were found to be more empowered than indigenous women [40]. This might be the possible explanation for non-indigenous women perceiving themselves as the the most influential person on decision-making processes. For the referral status result, emergency obstetrics incur higher health expenditures for referral cases where the decision makers were the husband or other family members [33, 41].

Poor to fair agreement was found between the women’s and their husbands’ responses on the most influential person for utilization of maternal health services. This result was similar to results from previous studies on spousal agreement in household decision-making, family planning and maternal health behaviours. The agreement on the issues of health decision making and the influential person are subjective and depends on the spousal communication [42, 43].

There were a few limitations in our study. First, the purposive sampling technique in our study may have reduced the representativeness of the study sample to the Nepalese population. Second, the samples were independent of type of delivery, area of admission (general ward or special cabin) and referral status, which might have influenced the woman’s decision to utilize the services. Third, the study involved facility-based data collection since this study aimed to identify the influential person of women who utilized the services. Thus, results cannot be generalized to pregnant women who do not utilize facility-based maternal health care services in Nepal. The respondents may have also felt the tendency of cultural norms on respecting their husbands and mothers-in-law, the so called autonomy effect, which may have overestimated the influence of other family members in decision-making, particularly among teens. However, all respondents were interviewed independently, thus the perception of social desirability was presumably less. Furthermore, the study subjects were forced to select the most influential person. This might have understated the role of other people who could have been jointly involved in the decision. The findings should be interpreted in the light of the context of Nepal where a safe delivery incentive program and national free delivery policy has been implemented which might have influenced the utilization of maternal health care services. Finally, we did not ask the subjects for the reason(s) why they ranked that person as the most influential on the woman’s decision to utilize the maternal health care services.

## Conclusion

Our study confirmed that both the woman and her husband had an influence on the decision to utilize maternal health care services based on the response from the woman herself, her husband and her mother-in law. It is evident that women in their teens and non-indigenous women are vulnerable groups and have a limited decision-making capacity on their utilization of maternal health services. The interventions for improving utilization of ANC and delivery care should be emphasized on the couple, especially among teen pregnant women and their husband.

Both women and their husbands influenced the decision to utilize ANC and delivery care. However, husbands were more influential than their wives, especially husbands of teens and young adults. Agreement of the perception on the most influential person between the women and their husband was less useful. Husband involvement is thus crucial in developing strategies to improve utilization of maternal health care services. Further studies on the perception of influential persons should be expanded to community-based settings.

## Authors’ original submitted files for images

Below are the links to the authors’ original submitted files for images. Authors’ original file for figure 1 Authors’ original file for figure 2 Authors’ original file for figure 3

## Abbreviations

ANC: Antenatal care
AOR: Adjusted odds ratio
CI: Confidence interval
MDG5: Millennium Development Goals 5
PMWH: Paropakar Maternity and Women’s Hospital
TUTH: Tribhuvan University Teaching Hospital
WHO: World Health Organization.
