# Supplementary figures and images for: Influence of family members on utilization of maternal health care services among teen and adult pregnant women in Kathmandu, Nepal: a cross sectional study
Source: Reprod Health. 2014 Dec 23;11:92. doi: 10.1186/1742-4755-11-92 (PMC4290463; doi:10.1186/1742-4755-11-92)

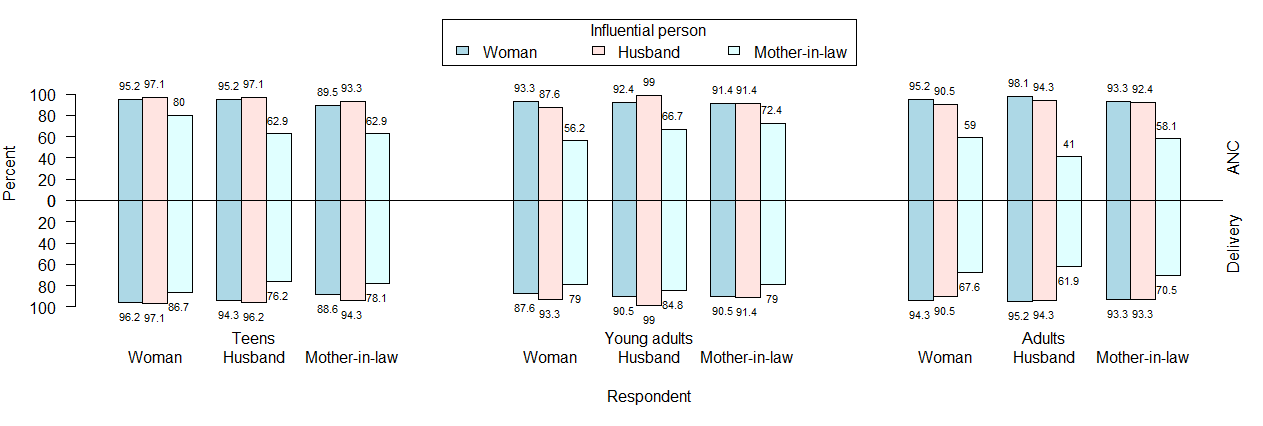

Supplement: Supplementary file 1 — Authors’ original file for figure 1 [file 12978_2014_336_MOESM1_ESM.png]

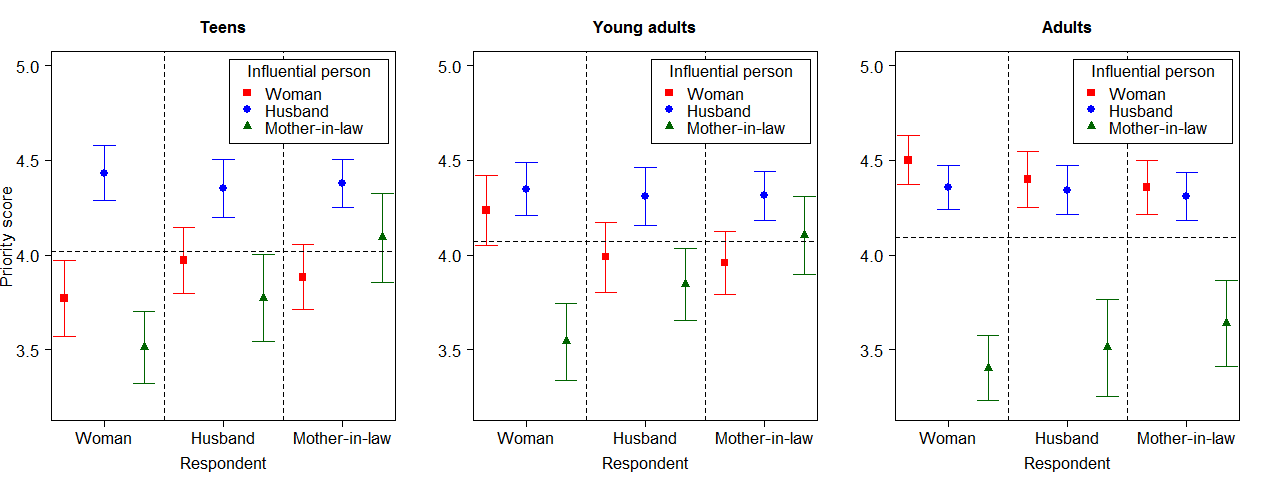

Supplement: Supplementary file 2 — Authors’ original file for figure 2 [file 12978_2014_336_MOESM2_ESM.png]

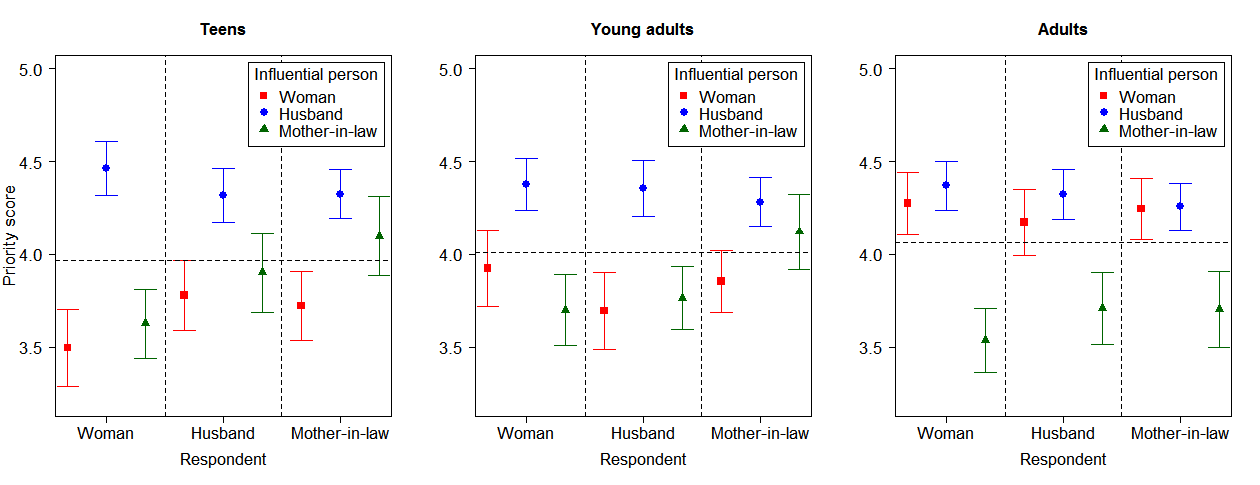

Supplement: Supplementary file 3 — Authors’ original file for figure 3 [file 12978_2014_336_MOESM3_ESM.png]
